# Chromosome Scale Assembly of Novel *Caenorhabditis* species #61 (JU4110)

**DOI:** 10.21203/rs.3.rs-9774402/v1

**Published:** 2026-05-22

**Authors:** Pooja Lad, Michelle A. McCauley, Victoria K. Eggers, Janna L. Fierst, Karolina Willicott

**Affiliations:** Florida International University; Florida International University; Florida International University; Florida International University; Florida International University

## Abstract

Species of genus *Caenorhabditis* tend to be similar morphologically but highly distinct on the genetic level, with recent characterizations being based on DNA sequences and mating systems. Among them, *Caenorhabditis elegans* has become a powerful model organism due to its short generation time, high reproductive output, and experimental amenability. However, beyond *C. elegans*, many *Caenorhabditis* species have yet to be elucidated; here, we report a chromosome-scale assembly of a novel *Caenorhabditis* species #61 (strain JU4110).

## Description

Despite the continually expanding genome resources for the Rhabditidae family of nematodes, few have chromosome-level assemblies and annotation. Many species of *Caenorhabditis* are highly understudied, including strain JU4110, also known as A325. This is a laboratory-derived, highly inbred line of *Caenorhabditis* species #61, produced from JU4045 through 25 rounds of single L4 female × single male crosses. JU4045 was originally isolated by the Marie-Anne Félix lab from a rotting flower near Sapa, Vietnam on November 30, 2019, and is characterized as a dioecious species.

We present a chromosome-level genome assembly and annotation for *Caenorhabditis* sp. 61, combining Pacific Biosystems (PacBio) HiFi long-read sequencing with Hi-C scaffolding. This paper aims to supplement existing *Caenorhabditis* genomic data and support future evolutionary and phylogenetic research.

## Methods

Prior to transfer to our research group, JU4110 remained cryopreserved. Standard *Caenorhabditis elegans* maintenance protocols were used to culture JU4110 populations on agar plates made from nematode growth media at 20°C, seeded with OP50 strain *E. coli*. To expand populations for DNA extractions, worms were transferred using a “chunk” of agar to three 100mm plates seeded with *E. coli* and were left to incubate at 20°C for 2–3 days. Mixed-age worms free of dauer larvae were washed off with M9 buffer into a 15mL conical tube and then washed twice with M9 buffer to reduce surface contaminants. Washed worms were finally resuspended in 10mL M9 and remained on a rocker overnight (~ 17 hours) to expel potential contaminants in the gut. Before extraction, two additional M9 washes were performed, worm bodies were isolated by centrifugation, the supernatant discarded, and the resulting pellet aliquoted into 50μL volumes in 1.5mL tubes.

### Long-read sequencing:

Promega Wizard^®^ HMW DNA Extraction Kit (cat. no. A2920) was used for DNA long-read sequencing using the manufacturer's protocol with minor modifications. Worm cuticles were disrupted through routine freeze/thaw cycles, alternating between − 80°C for five minutes and 37°C until thawed, with brief vortexing between cycles, repeated five times. All centrifugation steps were performed at 4°C, and alcohols were kept on ice until required. An additional incubation of 25 minutes at 65°C was incorporated at the lysis step. Samples were subsequently given to the University of Miami John P. Hussman Institute for Human Genomics Sequencing Core Facility (RRID:SCR_017828) for PacBio sequencing.

#### Hi-C

Extra tubes of 50μL worm pellet were frozen at − 80°C using a Mr. Frosty^™^ freezing container (Thermo Scientific cat. no. 5100–001) to prevent ice crystal formation. Two tubes were then mailed on dry ice to Arima Genomics for High Coverage Chromatin Conformation Capture sequencing (Hi-C).

#### Genome Assembly

Genome assembly was performed using Hifiasm v0.16.0 [6] with default parameters and PacBio HiFi and Arima Hi-C libraries. Contaminant contigs were later identified and discarded from both the diploid and phased haploid assemblies using BLAST v2.14.1 [4]. To identify duplications and remove alternative haplotypes, PacBio HiFi reads were mapped back to the assembly with minimap2 v2.30 using parameters -xasm5 -DP [14], after which read depth cutoffs were set manually using the -l 90 -m 114 -u 130 settings in purge_dups v1.2.6 [18]. At each step, assembly quality was assessed using QUAST v5.3.0 [15] with default parameters and BUSCO v6.0.0 [19] run against the Nematoda odb12 lineage dataset with options -m genome and --offline.

#### Hi-C Mapping

Hi-C raw data was processed and aligned with Juicer v2.0 [9] using default parameters and assembled using the --assembly option. We used YaHS v1.2.2 [28] for scaffolding and Juicebox v2.3.6 [22] for visualization and assessment.

### Gene and Repeat Annotation:

Genomes were first softmasked with RepeatModeler2 [14] and RepeatMasker [23], while RNA reads were aligned to the genome using STAR v2.6.1a with the -outSAMstrandField intronMotif option [8]. Genome annotation was subsequently performed using BRAKER3 v3.0.8 [11] on the softmasked assemblies using the Nematoda odb10 protein dataset with RNA sequence data from NCBI project PRJNA1256413 [20]. BRAKER3 depends on GeneMark (unsupervised) and AUGUSTUS (supervised), two generalized hidden Markov models for gene prediction [3, 24], with resulting protein sets consolidated by TSEBRA [12] to maximize BUSCO completeness. We subsequently filtered protein predictions for the longest isoform using AGAT v1.4.1 [7] via the scripts agat_sp_keep_longest_isoform.pl and agat_sp_extract_sequences.pl, and assembly statistics were generated with agat_sp_statistics.pl. Functional annotation was conducted with InterProScan v5.68.100.0 using the options -dp -goterms -pathways [16]. OrthoFinder v2.5.5 [10] was used to find single copy orthologs between JU4110 and *C. elegans*. The six major chromosomes were identified by location of single copy orthologs on *C. elegans* chromosomes. Nigon element classifications were assigned to single copy orthologs using a list of known gene:Nigon associations from [12].

Repetitive elements were identified and annotated using EarlGrey v6.0.1 [1] with options -r nematoda -e yes. EarlGrey applies a BLAST, Extract, Align, Trim (BEAT) process adapted from TEStrainer (https://github.com/jamesdgalbraith/TEstrainer), integrating several subprocesses including Tandem Repeat Finder [2], MREPS [17], SA-SSR [21], LTR_FINDER [26], RepeatModeler2, and RepeatMasker. Annotations were then merged and defragmented using RepeatCraft [25] for the final consensus repeat library.

#### Snail Plots

We used BlobTk v0.8.0 [5] to produce snail plots with the soft-masked assemblies. BUSCO v6.0.0 [19] and nematoda_odb 12 dataset helped recalculate assembly BUSCO scores.

## Figures and Tables

**Figure 1:**
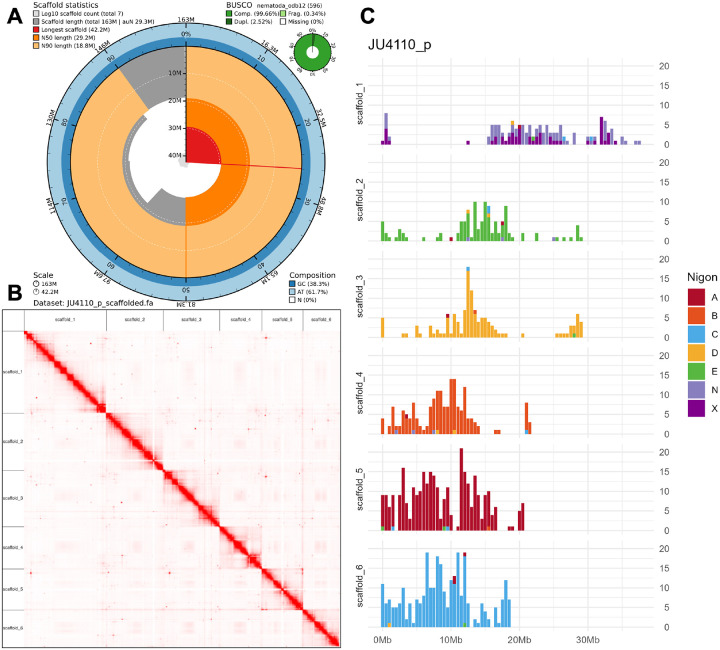
**A.** Snail plot (BlobTk) of assembly statistics for JU4110. The outer circumference represents the full length of the genome. The rings, moving from outermost to inner most describe various features: %GC content, N90 length, N50 length, longest scaffold, and scaffold length and number. The top right circle displays BUSCO scores. **B.** Hi-C contact map of JU4110 assembly reveals the six chromosome-scale scaffolds (X sex chromosome, autosomes 1–5, ordered from largest to smallest. Scaffold 7 is unplaced. **C.** Scaffolds with single copy orthologs to *C. elegans* genes previously assigned to Nigon elements (A-E, N, X) in 100kb bins.

**Table 1.** Properties of *Caenorhabditis* species #61

Location found	Sapa, Vietnam
Location coordinates	22.33947, 103.86148
Genome size (Mb)	163
Approximate coverage (x) – PacBio HiFi	119.0
Approximate coverage (x) – Illumina Hi-C	388.9
Number of reads (Gb) – PacBio HiFi	19.4
Number of reads (Gb) – Illumina Hi-C	63.4
GC content (%)	38.3
Number of protein coding genes	19,780
Repeats (%) – RepeatMasker	21.4
Repeats (%) – EarlGrey	26.5
SRA accession no. – PacBio HiFi	SRR38755789
SRA accession no. – Illumina Hi-C	SRR38755790
Isolated by	Marie-Anne Félix lab

## Data Availability

Bioinformatic scripts, workflows and software commands are available at https://github.com/jannafierst/HiC_Assemblies
